# Recent Developments on the Roles of Calcium Signals and Potential Therapy Targets in Cervical Cancer

**DOI:** 10.3390/cells11193003

**Published:** 2022-09-26

**Authors:** Jiahui Lei, Fengying Deng, Hongmei Ding, Mengyu Fu, Ting Xu, Bingyu Ji, Lin Feng, Min Li, Junlan Qiu, Qinqin Gao

**Affiliations:** 1 Institute for Fetology, The First Affiliated Hospital of Soochow University, Suzhou 215006, China; 2 Department of Obstetrics and Gynecology, First Affiliated Hospital of Soochow University, Suzhou 215006, China; 3 Department of Oncology and Hematology, Suzhou Science and Technology Town Hospital, Suzhou 215153, China

**Keywords:** cervical cancer, Ca^2+^ signals, occurrence, development, prognosis

## Abstract

Intracellular calcium (Ca^2+^) concentration ([Ca^2+^]i) is implicated in proliferation, invasion, and metastasis in cancerous tissues. A variety of oncologic therapies and some candidate drugs induce their antitumor effects (in part or in whole) through the modulation of [Ca^2+^]i. Cervical cancer is one of most common cancers among women worldwide. Recently, major research advances relating to the Ca^2+^ signals in cervical cancer are emerging. In this review, we comprehensively describe the current progress concerning the roles of Ca^2+^ signals in the occurrence, development, and prognosis of cervical cancer. It will enhance our understanding of the causative mechanism of Ca^2+^ signals in cervical cancer and thus provide new sights for identifying potential therapeutic targets for drug discovery.

## 1. Introduction

Intracellular calcium (Ca^2+^) is an important messenger that exists in almost all cells, and exerts biological effects through various signal pathways [1,2,3]. Increasing intracellular free Ca^2+^([Ca^2+^]i) is a universal mechanism of signal transduction, which controls a variety of cellular processes, including proliferation, metabolism, and gene transcription [1,2]. However, under certain conditions, the increase in [Ca^2+^]i concentration is cytotoxic. For example, intracellular Ca^2+^ overloading promotes apoptosis of myoblasts, and increasing intracellular Ca^2+^ can cause an escalation of reactive oxidative species [4,5]. The concentration of [Ca^2+^]i depends on various calcium channels, calcium pumps, and interacting proteins and ligands in the body to ensure its level within an appropriate range and give full play to its biological effects. Therefore, as a messenger, the concentration of [Ca^2+^]i is kept within a strict range [1]. 

Cervical cancer is the fourth most common cancer in women worldwide, the fourth highest cancer-related mortality rate in the world, and second only to breast cancer among cancers of the female reproductive system [6]. Among women, cervical cancer diagnoses accounted for 6.6% of all cancer types diagnosed during the same period, with a mortality rate of 7.5% [6]. Human papillomavirus (HPV, most notably HPV16 and HPV18) has been clearly defined as a human carcinogen, and its persistent infection in the cervix has been identified as an important cause of cervical cancer [7]. However, only a small part of persistent infection may develop into cervical cancer, which indicates that in addition to HPV, there are many other factors in the body that also contribute to the development of cervical cancer. Among them, the Ca^2+^ signaling pathway is closely related to the occurrence, development, and prognosis of cervical cancer [1,8]. Cellular Ca^2+^ homeostasis and signaling are maintained by a special set of proteins, such as Ca^2+^ channels, pumps, exchangers, sensors, and Ca^2+^ activation effectors [9]. When the expression, structure, and/or function of these proteins change, it may cause corresponding adverse effects. This review summarizes the latest findings regarding the pathogenic roles of Ca^2+^ channels, pumps and sensors, and Ca^2+^ activation effectors in cervical cancer, with the aim of providing new ideas and corresponding therapeutic targets for cervical cancer (Table 1).

## 2. Ca^2+^ Channels, Pumps, and Interacting Proteins and Ligands in Cervical Cancer

### 2.1. Transient Receptor Potential (TRP) Channels

TRP channels are a ubiquitous superfamily of ion channels in the body, responsible for a wide range of cellular functions, many of which regulate [Ca^2+^]i homeostasis and transduction [27,28,29]. TRP channels are gated by multiple stimuli, which differ between different family members. Some of them are triggered by mechanical stimuli and drive intracellular signaling pathways through spatiotemporally controlled Ca^2+^ influx [30]. Mechanosensitive Ca^2+^ channels play a key role in the rapid transmission of physical signals into biocompatible messages to influence key processes during development, morphogenesis, and regeneration [30]. TRP channels are divided into seven mammalian subfamilies according to their structural homology, including TRPV (vanilloid), TRPM (melastatin), TRPC (canonical), TRPP (polycystin), TRPML (mucolipin), TRPA (ankyrin), and TRPN (NOMPC-like) [27]. Each family has different activation modes [27,29,31]. As one of the channels for the influx of extracellular Ca^2+^, TRP channels play an important role in regulating [Ca^2+^]i concentrations [27]. Activation of TRP channels results in changes in [Ca^2+^]i concentrations, which are also required for the function of intracellular organelles, such as endosomes and lysosomes.

**TRPV1** could be gated by multiple stimuli, such as proinflammatory substances, heat, endocannabinoids, resiniferatoxin, lipoxygenase products, and peptide toxins [32]. TRPV1 mediated apoptosis mainly through protein signaling; in addition, this channel also stimulated proliferation through ATP release, purinoceptor 2 (P2Y2) activation, and epidermal growth factor receptor (EGFR) transactivation in [33]. Therefore, exogenous and endogenous TRPV1 agonists combined with P2Y2 and EGFR antagonists may constitute a potential form of anticancer therapy [33]. Some studies have found that the expression of TRPV1 in cervical cancer tissue is significantly higher than that in cervical intraepithelial neoplasia and normal epithelial tissue [10,11]. In cervical cancer tissue, the expression of TRPV1 was negatively correlated with the expression of PTEN (a suppressor gene) in [10]. High expression of TRPV1 was an independent prognostic factor for overall survival in cervical cancer in [10]. In particular, high TRPV1/low PTEN expression showed the highest hazard ratio for overall survival. In addition to this, the in vitro results of this study showed that overexpression of TRPV1 was associated with increased cell viability and colony formation, making it a potential candidate biomarker to predict the responses of cervical cancer cells to chemoradiation [10]. In addition, a study by Lucido CT et al. found that TRPV1 expression was significantly increased with the development of cervical cancer [11]. However, no relevant studies have confirmed whether the increased TRPV1 expression is associated with cervical cancer progression through pathways such as ATP release, P2Y2 activation, and EGFR transactivation. Based on existing research on the combined application of TRPV1 agonists and corresponding antagonists in cervical cancer and lung cancer cell [34,35], it is a very meaningful work to carry out TRPV1-related therapeutic targets for the treatment of cervical cancer. 

**TRPV6** is uniquely highly selective for Ca^2+^ and plays an important role in the extracellular Ca^2+^ influx pathway and the maintenance of Ca^2+^ homeostasis in organisms [30]. Ca^2+^ influx through this channel depends on intracellular and extracellular Ca^2+^ balance [36]. In addition to this, the activity of this channel can be regulated by hormones, such as estrogen and progesterone, tamoxifen, and vitamin D, leading to changes in cell proliferation and survival [37]. The relationship between TRPV6 and malignant transformation of various tissues has been extensively studied, and its expression level has been found to be elevated in many cancers, including breast cancer and pancreatic cancer [38,39]. The increased TRPV6 expression can stimulate cancer cell metastasis and generate chemoresistance [39,40]. In addition to this, high expression of TRPV6 is associated with cell proliferation and invasion through Ca^2+^-dependent pathways, and its elevated levels are thought to correlate with breast and prostate cancer prognosis [37,41,42]. However, in a study by Sun et al., it was found that both TRPV6 mRNA and protein levels were significantly reduced in early cervical squamous cell carcinoma tissues and cell lines [12]. The expression of TRPV6 in early-stage cervical cancer was significantly correlated with the tumor stage, tumor growth type, tumor size, differentiation grade, and poor prognosis [12]. Moreover, the early-stage cervical cancer patients with a low TRPV6 expression had a short progress-free survival and overall survival duration [12]. Univariate and multivariate analyses identified TRPV6 as an independent prognostic factor for early cervical cancer patients’ survival, indicating that TRPV6 may be used as a novel prognostic marker for early cervical cancer [12]. The expression of TRPV6 in cervical cancer is different from other cancers, and additional research will be needed to validate this difference, as well as to uncover the underlying mechanisms and develop novel therapeutic targets based on TRPV6.

**TRPM4** belongs to the TRPM channel subfamily. Unlike other TRP families, it does not directly transduce Ca^2+^, but is directly activated by intracellular Ca^2+^, which then directs Na^+^ influx. The influx of Na^+^ depolarizes the plasma membrane, reducing the driving force of store-operated Ca^2+^ entry (SOCE) and other Ca^2+^ entry pathways, thereby regulating [Ca^2+^]i concentrations [43,44]. Although TRPM4 is impermeable to Ca^2+^, Na^+^ influx through TRPM4 reduces membrane potential and leads to a reduction in Ca^2+^ signaling in many different cells, including cancer cells [31]. Thus far, TRPM4 has been investigated in various cancers. In several types of cancer, including colorectal tumor and prostate cancer, TRPM4 is overexpressed and contributes to cancer hallmark functions, such as proliferation, migration, invasion, and the epithelial-to-mesenchymal transition [45,46]. Hence, TRPM4 is considered a potential diagnostic marker for cancer progression and a promising anticancer drug target candidate [47]. A gene expression study of cervical cancer cases reported that TRPM4 was overexpressed in cervical cancer specimens compared with normal cervical epithelium [13]. Hong et al. reported that prostate cancer cells with reduced TRPM4 expression were found to have changes in cell distribution in each cell cycle [14]. Consistent with the study on prostate cancer cells, knockdown of TRPM4 in the cervical cancer-derived cell line HeLa also caused corresponding cell cycle changes, thereby reducing the proliferation of HeLa cells via β-catenin degradation [15]. This study of the cell cycle distribution of shRNA-mediated downregulation of TRMP4 levels found a significant reduction in the S-phase cell population, but a significant increase in the G1 phase cell population [15]. Moreover, after TRPM4 gene knockout, the expression levels of cell-cycle-related proteins changed, such as the decreased expression level of cyclin D1. Cyclin D1 promotes the transition of cells from G1 to S phase, which may explain the decreased cell proliferation observed after TRPM4 knockout [14]. TRPM4 is a potential prognostic cancer marker; further studies using TRPM4-KO cells or a specific TRPM4 inhibitor (9-phenanthrol, flufenamic acid, NBA (2-(1-naphthyloxyacetamido)-4-chloro -benzoic acid), and LBA (4-chloro-2-(2-(4-chloro-2-methylphenoxy) propanamidei) benzoic acid)), CBA ((4-chloro-2-(2-chlorophenoxy) acetamido) benzoic acid, also called compound 5)) treatments could give a better overview of the role of TRPM4 in the development, growth, and metastasis of cervical cancer [31,48]. Furthermore, based on the fact that TRPM4 is a channel located on the cell membrane, it will be an interesting target on inhibiting proliferation of cervical cancer cells [31].

**TRPM7** preferentially allows the flow of Mg^2+^ and Ca^2+^, and in some cell types, the influx of Mg^2+^ through TRPM7 channels can lead to changes in intracellular Ca^2+^ levels [49,50]. Accumulating evidence has shown that TRPM7 is aberrantly expressed and/or activated in human cancers. TRPM7 plays a variety of functional roles in the hallmarks of cancer, including survival, cell cycle progression, proliferation, migration, and invasion [51,52]. More and more data also show that TRPM7 has potential value as a molecular biomarker and therapeutic target for human malignant tumors [52]. TRPM7 is also found to be aberrantly overexpressed and/or activated in cervical cancer, and has been discovered as the direct therapeutic target for cervical cancer [16,17,18]. For instance, Liu et al. demonstrated that TRPM7 is a target of miR-543 (a class of miRNAs that play an important role in the occurrence and development of various human carcinogenesis) in cervical cancer [16]. Restoration of TRPM7 expression partially reversed the tumor suppressive role of miR-543 on cervical cancer progression by promoting cell proliferation and invasion and attenuating cell apoptosis [53]. Dong et al. reported that miR-192-5p (a miRNA with broad anticancer effects) performs an inhibitory role in cervical cancer proliferation and invasion by targeting TRPM7 [17]. In addition, TRPM7 channel activity has proven to play an important role in the death of necrotic volume increase and necrotic cell in the acid poisoning of HeLa cells. Progesterone can inhibit the expression and activity of TRPM7, thereby transforming acid poisoning cells from necrotic to apoptosis [54].

**TRPC** is a classic transient receptor potential channel in the body. It generally comprises of seven isoforms, labeled TRPC1-7. These channels can regulate Ca^2+^ balance and promote cell cycle regulation and the expression/activation of Ca^2+^ related factors, thereby playing a key role in cancer cell proliferation [55,56]. Studies have found that the expression of TRPC in breast cancer tissues has increased significantly [57]. TRPC can be used as a potential drug target for cancer diagnosis and treatment [57,58]. However, there is no related research on the relationship between this channel and cervical cancer.

Apart from these, there are many TRP channels that are associate with intracellular Ca^2+^ concentration in cancer. However, there are no relevant studies to confirm their relationship with cervical cancer. Follow-up related research can be carried out, and then find out the target of cervical cancer treatment related to these TRP channels.

### 2.2. Ca^2+^ Release-Activated Ca^2+^ (CRAC) Channel

Store-operated calcium entry (SOCE) through the CRAC channel is a central mechanism by which cells generate Ca^2+^ signals and mediate Ca^2+^-dependent gene expression. The CRAC channel, composed of Ca^2+^ release-activated Ca^2+^ channel protein 1 (Orai1) and stromal interaction molecule (STIM), represents a prototypical example of SOCE to mediate Ca^2+^ entry between the endoplasmic reticulum and the plasma membrane in most nonexcitable cells [59,60]. There is accumulating evidence to indicate that the CRAC channel can influence various processes associated with tumorigenesis [61,62]. Abnormal expression of CRAC channel proteins has been observed in several types of cancer cells, indicating that CRAC-channel-activated Ca^2+^ influx will be a potential therapeutic target for cancer [61,62]. STIM recognizes a signal of reduced [Ca^2+^] in the ER and transmits this signal to Orai1 channels in the cell surface membrane to promote Ca^2+^ influx. In cancer cells, SOCE plays an important role in the cell cycle process, proliferation, migration, metastasis, and evasion of apoptosis [63]. The changes in the expression of a key element of reshaping SOCE and Ca^2+^ steady state play an important role in the transformation of the phenotypes observed in the transformation cell [64].

In cervical cancer, a recent study found that histone deacetylase 6 (HDAC6) is required for STIM1 translocation on microtubules and thus activates Orai1-mediated SOCE [19]. The levels of Orai1, STIM1, and HDAC6 were upregulated in cervical cancer cells [19]. Inactivation of HDAC6 with drug inhibitors or molecular knockdowns leads to low acetylation of tubulin and SOCE abolition. Interestingly, the expression of STIM1 and Orai1 was increased in most cervical cancer specimens, while the acetylation of tubulin was decreased [19,20], suggesting that specific targeting of HDAC6 in cervical cancer can inhibit STIM1-Orai1-mediated cervical neogenesis. STIM1 is very important for cervical cancer cell proliferation, migration, and vascular generation [19]. Increasing STIM1 expression is related to increased metastasis and decreased survival. STIM1 silencing in cervical cancer cells can significantly inhibit cancer cell proliferation. The excessive expression of STIM1 enhances the invasion of cervical cancer cells, while the knockout of STIM1 weakens this migration [19,20]. In addition, STIM1 can regulate the secretion of VEGF-A by cancer cells [20]. STIM1′s expression in tumors is also closely related to the clinical prognosis of early cervical cancer [20]. Related studies have found that SOCE inhibitors have the effect of blocking tumor blood supply in cervical cancer, prostate cancer, and breast cancer [20,65,66]. The SOCE channel and its related protein-mediated Ca^2+^ signal transformation is of great significance for the biological effects of the cell. Therefore, deeper research on SOCE will help to better understand the occurrence, development, and prognosis of cervical cancer, contributing to the development of novel methods and targets for the treatment of cervical cancer.

### 2.3. Inositol 1,4,5-Triphosphate Receptor (IP3R) and Ryanodine Receptors (RyR) Channels

Intracellular Ca^2+^ release channels in ER consist of a subset: mainly including IP3R channels and RyR channels. IP3R channels are activated by IP3 binding, and RyR channels are activated by elevated [Ca^2+^]i or protein signaling and ER releases Ca^2+^ through these channels [67,68].

The IP3R/Ca^2+^ signaling pathway is an extremely important part of maintaining body homeostasis. The signaling pathway has direct and indirect effects on the action of cells, including control of cell metabolism, secretion, fertilization, proliferation, and smooth muscle contraction [69,70]. Changes in IP3R/Ca^2+^ signaling are important factors in the development of a large number of human diseases [71]. Increasing evidence has shown that changes in the IP3R/Ca^2+^ signaling system are responsible for altered Ca^2+^ signaling in many cancer cells, such as lung cancer and cholangiocarcinoma clear cell renal cell carcinoma, and are closely related to the occurrence, development, and prognosis of cancer [72,73,74]. There are three IP3R isoforms—IP3R1, IP3R2, and IP3R3—expressed in mammals in different amounts. Yang et, al. reported that IP3R3 genetic polymorphisms are associated with the risk of cervical cancer [21]. The ITPKC gene encodes inositol 1,4,5-trisphosphate 3-kinase C, which inactivates IP3R3. ITPKC can inhibit the IP3 pathway by phosphorylating the active ligand of IP3Rs, inositol-1,4,5-trisphosphate (IP3), to a less active/inactive form (inositol-1,3,4,5-tetrakisphosphate), thereby weakening the Ca^2+^ signaling pathway, and its genetic polymorphism is associated with an increased risk of cervical squamous cell carcinoma [22,75].

RyR channels are the largest known ion channels that are located in the membrane of the ER and are expressed in a restricted subset of cell subtypes, such as cardiac and skeletal muscle cells [76]. One of its important roles is the release of Ca^2+^ from intracellular stores during excitation–contraction coupling in cardiac and skeletal muscle [77]. RyRs have three isoforms, RyR1, RyR2, and RyR3. They are embedded in the ER, mediating intracellular Ca^2+^ release, thus leading to the generation of a quick, transient increase in cytosolic Ca^2+^ levels [78]. RyRs are known to play key roles in the control of some major biological processes, such as metabolism, cell proliferation, and apoptosis [78]. RyRs can also be involved in the occurrence and development of cancers, including ovarian cancer, head and neck cancer, and prostate cancer [79,80,81]. Schmitt et al. indicated that impaired RyR2 function by either somatic mutation or epigenetic silencing is a common event in head and neck squamous cell carcinoma pathogenesis. Detection of RyR2 expression may be useful in assessing the risk of malignant transformation in dysplastic lesions [79]. Abdul et al. indicated that RyRs expression correlates with tumor grade in breast cancer; RyRs could serve as a prognostic indicator and/or as a target for breast cancer treatment [82]. Law et al. revealed the cytotoxic mechanism of neferine-induced autophagy through RyRs activation in resistant cancers, providing insights into the exploitation of novel interventions based on RyRs [83]. However, there are few studies on the relationship between the occurrence, development, and prognosis of cervical cancer and RyRs/Ca^2+^ signaling. Such studies cannot conclusively provide ideas for obtaining corresponding therapeutic targets.

### 2.4. SERCA Pump

Calcium is actively accumulated in the ER by sarcoendoplasmic reticulum (SR) calcium transport ATPase (SERCA enzymes). Unlike the above-mentioned channels that regulate ER Ca^2+^ homeostasis by releasing Ca^2+^, SERCA-dependent Ca^2+^ transport is the only Ca^2+^ uptake mechanism in this organelle [9]. Therefore, regulation of SERCA function is a key mechanism for regulating ER Ca^2+^ homeostasis according to cell type and its differentiation state. Regulation of SERCA activity can affect cell differentiation and survival [9]. Three SERCA genes are known (SERCA1, SERCA2, and SERCA3) by alternative splicing. The expression level of SERCA changes significantly during cell differentiation or tumorigenesis, resulting in altered ER calcium storage [9,84]. Studies have shown that elevated SERCA2 expression was detected in malignant cervical cancer, and this change was positively correlated with the clinical stage of malignant cervical cancer [23]. SBF-1, a synthetic steroidal glycoside, binds directly to SERCA2 to inhibit its function [85]. In human cervical cancer cells, SBF-1 represses SERCA2 function both in vitro and in vivo, thereby disrupting ER Ca^2+^ homeostasis and inducing ER stress-mediated cancer cell death. The study also found that SBF-1 inhibited the growth and migration of HeLa cells depending on the activity and level of SERCA2 [23]. In addition to this, they also indicated that SERCA2 is a potential therapeutic target for human cervical cancer. The concept of using SBF-1 as a chemotherapeutic drug in the treatment of cervical cancer is interesting. However, the application of SBF-1 is very limited due to its immunosuppressive activity on T lymphocytes and its possible adverse cardiovascular effects by affecting cell-wide SERCA2 channels [86].

### 2.5. Mitochondrial Calcium Uniporter (MCU) Channel

MCU is the pore-forming subunit of the MCU complex, which consists of MCU, the scaffold protein EMRE (*Escherichia coli* efflux-multidrug resistance E), and the Ca^2+^ sensitive inhibitory regulatory subunits MICU1 and MICU2 [87]. The MCU channel and its associated regulators transport Ca^2+^ across the inner mitochondrial membrane to the mitochondrial matrix. Due to this central role and ability to influence cell behavior and fate, several research groups are investigating the role of the MCU complex in different cancers and cancer-related diseases [87,88,89]. Currently, there have been very limited data on the changes of MCU expression or activity in cervical cancer. In one study, it was found that siRNA silencing of MCU in HeLa cervical cancer cells significantly reduced mitochondrial Ca^2+^ uptake. Furthermore, in HeLa cervical cancer cells, overexpression of MCU or knockdown of MICU1 resulted in constitutive mitochondrial Ca^2+^ influx [90].

### 2.6. Others

There are other molecules related to Ca^2+^ signaling in the body, such as S100 calcium-binding protein. When S100 calcium-binding protein binds to Ca^2+^ and can change its structure and function, thereby acting as a Ca^2+^ sensor, translating fluctuations in intracellular Ca^2+^ levels into cellular responses [91,92]. There are many subtypes of S100 calcium-binding protein, and several studies have found that S100A7, S100A9, S100A11, and S100A14 are closely related to cervical cancer [24,25,93,94]. Studies have shown that the expression of S100A11 in cervical cancer tissue is significantly higher than that in paracancerous tissue and normal cervical tissue. The overexpression of S100A11 can promote the proliferation and metastasis of cervical cancer cells [24]. Wang et al. found that the expression of S100A14 was increased in cervical cancer tissues, and this increase in expression was closely related to whether cervical cancer had metastasis [25]. They also found that in cervical cancer cells, S100A14 overexpression increased the ratio of the G2/M phase, which in turn promoted cell proliferation, migration, and invasion. The S100A14 gene knockout can eliminate the above changes [25]. Similar to S100A14, the expression of S100A7 was also significantly upregulated in cervical cancer tissues compared with normal cervical tissues. There is a correlation between S100A7 expression and tumor grade and lymph node metastasis [93]. In addition, S100A9 is overexpressed in cervical cancer [94]. The above S100 proteins are all related to the proliferation, metastasis, and invasion of cervical cancer. However, whether these pathological processes are realized through the Ca^2+^ pathway has not been confirmed yet.

The intermediate-conductance calcium-activated potassium channels (IKCa1) functions are strictly dependent on Ca^2+^. IKCa1 are expressed in many tissues and play a variety of physiological roles, including regulation of intracellular Ca^2+^ homeostasis [95,96,97]. In normal cells, IKCa1 regulate K^+^ efflux and hyperpolarization after channel activation by releasing intracellular Ca^2+^ stores [98]. In addition to this, IKCa1 are involved in cell proliferation. Several studies have shown that the functions of IKCa1 are required for Ca^2+^-sensing steps in cell cycle progression. Cells in a hyperpolarized state exhibit enhanced Ca^2+^ entry and Ca^2+^ homeostasis through K^+^ channel activation, which are critical for controlling cells through the G0/G1 or G1/S phase transition [99,100]. Related studies have found that drug blockade or gene reduction of IKCa1 in vitro reduced pancreatic and hepatocellular carcinoma cell proliferation and inhibited cell growth [101,102,103]. Ling L. et al. found that IKCa1 are highly expressed in cervical cancer tissue; the higher the malignancy of cervical cancer tissue has, the higher the IKCa1 expression is [26]. The findings also suggest that IKCa1 are involved in promoting the dedifferentiation and proliferation of malignant cervical cancer cells [26]. When using clotrimazole (an IKCa1 blocker), or knocking down IKCa1 using siRNA, the decrease in IKCa1 currents reduces the ability of Ca^2+^ influx, which reduces intracellular Ca^2+^ signaling that regulates cell cycle progression [26]. It further leads to the increased apoptosis of cancer cells, thereby inhibiting the growth and proliferation of cervical cancer HeLa cells [26].

## 3. Summary and Outlook

**Figure 1:**
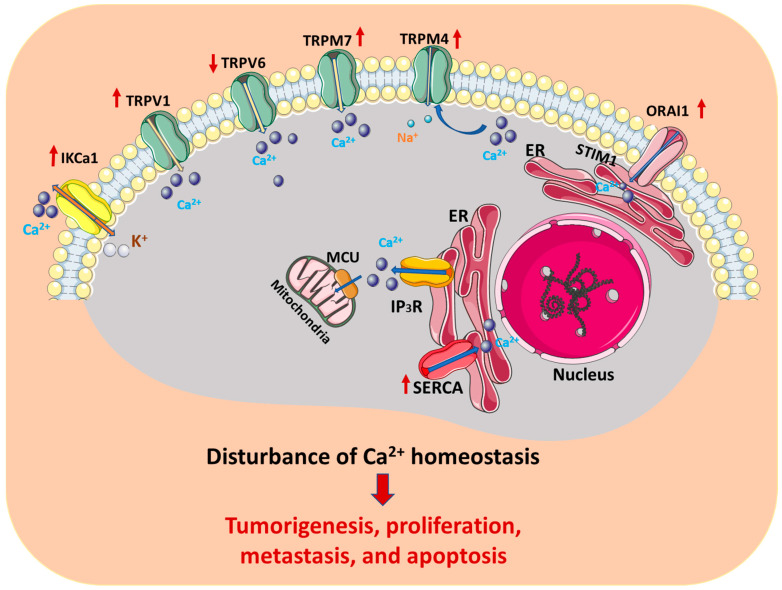
Ca^2+^ channels/pumps/exchangers in cervical cancer. Ca^2+^ channels/pumps/exchangers play an important role in affecting the concentration of Ca^2+^ inside and outside the cell: (1) plasm membrane Ca^2+^ channels or transporters, including IKCa1, TRPs, and CRACs; (2) intracellular calcium release channels in ER mainly include IP3R channels; (3) SERCA-dependent Ca^2+^ transport is a Ca^2+^ uptake mechanism in ER. Changes in Ca^2+^ channels/pumps/exchangers related to the regulation of Ca^2+^ homeostasis could affect cervical cell metabolism, proliferation, and apoptosis, and lead to the development of cervical cancer. IKCa1: intermediate-conductance calcium-activated potassium channels; ER: endoplasmic reticulum; TRPs: transient receptor potential channels; MCU: mitochondrial calcium uniporter; IP3R: inositol 1,4,5-triphosphate receptor; SERCA: sarcoendoplasmic reticulum calcium transport ATPase; STIM1: stromal interaction molecule 1.

Through previous studies on the relationship between Ca^2+^ signaling and cervical cancer, we can easily find that Ca^2+^ signaling plays an important role in cervical cancer growth, proliferation, and metastasis. Changes in various Ca^2+^ channels, pumps, exchangers, sensors, and Ca^2+^ activation effectors related to the regulation of Ca^2+^ homeostasis can affect the concentration of Ca^2+^ inside and outside the cell, thereby affecting cervical cell metabolism, proliferation, and apoptosis, and eventually leading to the development of cervical cancer (Figure 1).

In addition to the Ca^2+^ channels, pumps, and interacting proteins and ligands mentioned above, there are still many Ca^2+^-regulatory factors that regulate Ca^2+^ homeostasis and signal transduction related to the occurrence of cancer in vivo. For example, studies have shown that Orai3 is involved in various processes of breast cancer, such as proliferation and survival and resistance to chemotherapy, and the expressions of secretory pathway Ca^2+^-ATPases (SPCA1) and SPCA2 genes are significantly increased in breast cancer [104,105]. The plasma membrane Ca^2+^-ATPase (PMCA) is ubiquitously expressed, which is critical for maintaining low resting cytosolic Ca^2+^ in cells. Studies spanning many years have revealed that some cancers are associated with a remodeling of PMCA expression [106]. The sodium/calcium exchanger (NCX) and the therapeutic potential of its inhibitors have been also studied in cancer [107]. Piezo1, a mechanically activated ion channel, can regulate Ca^2+^-dependent signaling cascades associated with tumor cell migration by promoting local Ca^2+^ influx [108]. Piezo1 is involved in colon cancer cell metastasis; its expression is significantly elevated in prostate cancer cell lines and human prostate cancer tissues, and downregulation of Piezo1 significantly inhibits prostate cancer cell survival, proliferation, and migration [108,109]. In addition, some studies have also found that the multidrug resistance of cancer cells can be overcome by regulating the T-type Ca^2+^ channel [110].

However, there is no relevant research to show whether these channels or protein- regulated Ca^2+^ homeostasis and signal transduction are related to the occurrence, development, and prognosis of cervical cancer. Research findings in other tumors may provide new ideas for cervical cancer research. Existing studies on Ca^2+^ signaling related to cervical cancer treatment are still few, and the current research has not included Ca^2+^ signaling targets as the focus of cervical cancer treatment. Increasing expression or silencing of some Ca^2+^ channel proteins related to tumor cell apoptosis has been confirmed in the treatment of other tumors, which provides a new idea for the targeted therapy of cervical cancer. Admittedly, little is known about the effect of Ca^2+^ signaling in cervical cancer; there is, thus, a need for additional research in this field.

## Figures and Tables

**Table 1:** Ca^2+^ channels/pumps in cervical cancer.

Ca^2+^ Channels/Pumps	Expression	Effects on Cervical Cancer	References
**TRPV1**	Increased	Increased hazard ratio for overall survival, cell viability, and colony formation	[10,11]
**TRPV6**	Decreased	Lead to poor prognosis	[12]
**TRPM4**	Increased	Promote cancer cell proliferation	[13,14,15]
**TRPM7**	Increased	Promote cancer cell proliferation and invasion	[16,17,18]
**Orai1 and STIM1**	Increased	Cancer cell proliferation, migration, and angiogenesis increase; correlates with prognosis	[19,20]
**IP3R3/ITPKC**	/	Genetic polymorphism is associated with an increased risk of cervical squamous cell carcinoma	[21,22]
**SERCA2**	Increased	Positive correlation with clinical stage	[23]
**S100A7, S100A9, S100A11, S100A14**	Increased	Correlation with tumor grade and lymph node metastasis; promotes cancer cell proliferation, migration, and invasion	[24,25]
**IKCa1**	Increased	Positively correlated with malignancy, promoting dedifferentiation and cancer cell proliferation	[26]

## Data Availability

Not applicable.
